# Notes from Private Practice

**Published:** 1875-09

**Authors:** J. G. Thomas

**Affiliations:** Savannah, Ga.


### NOTES FROM PRIVATE PRACTICE.

By J. G. THOMAS, M.D., Savannah, Ga.

### A CASE OF SYPHILITIC FEVER.

Was summoned 12th May to a hotel, to see Mr. L., who had just landed off steamship from New York; had been three days and a half on water. He gave me the following history of his case: The preceding March 24th discovered a slight pimple on the glans and frænum of the penis, and being in B——, South Carolina, called upon a well-known physician, who pronounced it "simply nothing," "not even a chancroid." Might have said it was herpes. April 3d, being in D——, North Carolina, and the sore still unhealed, called on Dr. S., who, after examination, pronounced it "chancre." It was then a small ulcer, principally upon frænum. Dr. S., regarding it as syphilis, proposed to treat it as such: but Mr. L., being in doubt, and believing that it had been increased by traveling, and having business in another city of North Carolina, after allowing the doctor to cauterize it once, went on to F——, where he saw Dr. A., April 5th. There were now four distinct ulcers, two on the glans and two on the prepuce and frænum. The latter doctor said it was "not chancre," "possibly chancroid," "more likely a mere local sore." From here Mr. L.'s business called him to Philadelphia, where he consulted Dr. R. By this time the sores were nearly healed, but both groins were considerably swollen. On the first examination Dr. R. pronounced it chancre, but when he told him that he was liable to swollen glans, he changed his opinion, and said it was chancroid. The glands continued to swell for several days, and were very much aggravated by a trip to New York, where urgent business called him, and where he went against the advice of the physician. On his return, walking was very painful, the groins very much swollen, and he had a slight fever. Dr. R. treated the buboes with compressed sponge, bandages and hot water. There was no suppuration. Had slight fever, off and on, up to the 8th May, when he left Philadelphia for New York, to sail for Savannah. Glandular swelling entirely gone down, though some soreness on motion. Left on steamship afternoon of 8th, and being a good deal on deck, and a raw northeaster prevailing, thinks he caught cold. Fever came on decidedly on the 10th, with sore throat.

This is almost the verbatim account that he gave me on my first visit. When I first saw him he had decided fever, the thermometer indicating $102\frac{3}{4}$; pulse full and strong at 100; no cough, throat very much inflamed and having the appearance of a severe catarrh. Bowels regular, urine highly colored. In the course of the examination I casually asked if he was now entirely satisfied that his case had not been syphilis, and he answered promptly, "perfectly so." After examination of lungs, heart, etc., and finding nothing in the various organs to account for the fever, prescribed quinia thirty grains, in five-grain doses every two hours, seidlitz powder to open the bowels, and choral if he could not sleep.

Saw him again on the morning of 13th, and found the temperature had gone down to $101\frac{3}{4}$, and decidedly nervous, restless and depressed. His depression, he said, was owing to my question the previous day, whether his mind was at rest about the syphilis. His throat continued to grow worse until he was unable to swallow anything except equal parts of strong black tea and cream, or a little thin sago and milk. From this time for four or five weeks his temperature ran from 101 to 104; not often getting as low as the former and very frequently going as high as the latter in the evenings. He was, of course, losing strength all the time, and his mind often wandered during the height of the fever. The case annoyed me much to know exactly what to call it. It was not typhoid, for, except the fever, which was mere remittent, there was not a typhoid symptom. Quinine would lower the temperature, as it does in all febrile exacerbations, but it would not arrest it, and in a day or so it would be as high as ever. Many thorough physical examinations satisfied me that there were no organs involved sufficiently to account for this high grade of fever; and previous to this I had never seen so high and persistent a fever from syphilis.

On 4th June I asked Dr. Harriss to see the case with me, and think he was as much puzzled as I had been to account for the fever.

June 15th, severe pains in limbs, particularly at night. His throat still quite sore, though better than it had been, from repeated applications of solution nitrate silver, carbolic acid and glycerine, solution permanganate potass, etc.

18th June, temperatnre $102\frac{1}{2}$ in the early morning, and in the evening it was 104. It continued at this high rate for three days, and we felt that something must be done, or else our patient would succumb. It was agreed that we should try to reduce the fever by cold water. The patient readily consented, for he said the fever was absolutely burning him up. An ordinary bath-tub was obtained, long enough to submerge the entire person up to the neck, and placing it by the bedside, we had it filled with hydrant water, temperature 80. Took temperature of patient just as he went into the bath, and it was 104. Had a thermometer placed in his mouth, and watched it for half an hour with very little reduction. Sent off for fifty pounds of ice, and ordered five pounds at a time to be put into the water, and to watch the thermometer. All of this and twenty-five pounds more were consumed before the heat began to yield; when quite rapidly it fell down to 97. It fell, however, two degrees in the time consumed in taking him out and getting him snugly wrapped in a blanket in bed. He had a slight rigor after he was put to bed; but in one hour he said he had not felt so well in weeks. His temperature did not go above 99 during the night. Next day by eleven o'clock his temperature was 100, and he ordered himself the bath to be prepared. About 3 P.M., when it had gone up to 102½, I allowed him to get into it again. This time thirty minutes sufficed to reduce the heat to 98, and he had no rigor. After this he was able with our hydrant water at 80 to take his fever off when ever he chose.

July 1st; A few pimples began to appear on his face, neck and arms, and in three or four days he had a blooming case of syphilitic eruption, both papular and pustular, and a goodly number of sores about his penis, which, however, had appeared a week previously.

Baumler, in his article on syphilis, in Zeimssen's Cyclopædia of Practice, vol. iii., page 126, says "that the syphilitic fever commences most commonly between fifty and sixty-five days from the period of infection; the period may also be deferred to the ninetieth day." He says, also, "the fever may consist of a sudden, marked elevation of temperature, occurring but once, or it may continue for days, during which time the temperature may reach nearly 104, and then fall rapidly again. This recession of fever is only temporary, however, and merges into remittent fever of moderate grade, which may last for weeks." He says also that as in other infectious diseases, the fever precedes the eruption, and that it runs in a lower grade or entirely disappears after the eruption appears." He intimates that the fever has been mistaken for remittent as well as for typhus.

There appears to be two or three striking peculiarities about this case which prompted its publication.

Firstly. The very high grade of fever, which reached a temperature of 104 on more than one day, and its very obstinate and persistent type, giving evidence unless arrested it would soon put an end to the life of the patient.

Secondly. The very great benefit he received under the reductive treatment of cold water; also the absolutely *iced bath* that it required at first to reduce the temperature, and the entire control that he had over it afterwards with a very moderate bath.

Thirdly. The eruption did not begin to appear until nine days after the use of the bath was begun, and therefore the appearance of the eruption had nothing to do, as is claimed by others, with the decline of the fever.

Fourthly. The uncertainty which there seemed to be about the primary sores. I am inclined, as far as my knowledge goes, to be a believer in the duality doctrine, but am under the belief also that great injury has befallen more than one case where good physicians have seemingly been over confident that they had a "chancroid" to deal with, and assured their patients that there was no necessity for either anxiety or constitutional treatment. More than one such case has fallen under my care that have had such assurances given them, and where they have been entirely lulled into doing nothing until five or six weeks had elapsed, and their hair began to drop, and other indications of secondary trouble began to appear. This certainly is unfortunate. These mistakes, too, are not confined to the mediocre men in the profession, or else we might attribute it to ignorance, (and I am not fully prepared to say that it was not ignorance of what they ought to have known in some of the cases); but it seems that there is yet great doubt hanging over these primary sores, which we are either careless in giving our opinions upon, or else there are sores which will perplex the best observers. My own impression is that the latter is the true interpretation to put upon such facts.

My case as it stands now is a well developed case of secondary syphilis.

A CASE OF ABSCESS OF THE LEFT LOBE OF THE LIVER, DISCHARGED THROUGH THE ASPIRATOR. RECOVERY.

On the 15th of May I was called to see Willie Spencer, a child of seven years of age, with what was supposed to be a "boil on the side." I found a large abscess *outside* of the ribs, on the right lower edge of the right hypocondrium. Careful examination satisfied me that it was external to the ribs. I opened it and it discharged a couple of gills of laudable pus. As he had given an account of a "fall against the fence," striking his right side, I was careful to examine after the discharge of the pus whether the ribs were injured, and whether there was any connection internally with the abscess, but found neither. The place healed, and to all appearances the boy recovered at once.

In two or three weeks I was sent for to see him again, suffering from constant fevers. There was slight fever, with dry skin, quick pulse, temperature 101½. On percussion, found decided tenderness over left lobe of the liver, several inches to the left of the previous cut, which had entirely healed, leaving only a little discolored blotch, with eschar. Diagnosis—inflammation of left lobe of the liver. Next day fever as day before, with increased tenderness around in the arch under the ensiform cartilage. There was a decided doughy feeling about the region. These symptoms satisfying me that there would be a formation of pus underneath, I decided to wait six or seven days to be sure of the pus formation, and puncture with aspirator.

On the eighth day, being convinced from the increase of all the foregoing symptoms, with the kindly assistance of Drs. Harriss and Duncan, using the latter's instrument, I pushed the second sized trocar of Dieulafoy's aspirator three inches into the central part of the swelling, just two inches and a half under the ensiform cartilage, and after freeing the end of the trocar we turned the stop-cock, when a gill of pus spirted into the receiver. In a week's time the little fellow was playing about the floor, and had no fever from the date of operation.

This case, as I conceive, has two points of interest to physicians. The first is the diagnosis; the second is the value of this instrument, but for which I doubt if this child's life could have been saved.

Note.—The treatment above spoken of for the cure of syphilitc fever, I have been pursuing very considerably in other forms of fever, and am much pleased with it. We have had this summer considerable bowel trouble amongst children; some cases going as far as cholera infantum. In most of the cases I found a temperature of 102, and from this a few going as high as 105. There is great restlessness, and often nothing stays on the stomach. Such cases as the latter particularly I put into a bath of about 90 or 93, and then gradually reduce the temperature of the water to 80 or 85; and in the majority of cases on the first introduction the child's temperature will fall to 98 and even a little below in twenty or thirty minutes, and, taking it out, put it to bed, and it will drop into a long sleep, and wake up refreshed.

This I do daily, and have been doing it the whole of this summer. There is, as I know, nothing very novel in this treatment, but I do not know of its being very generally followed in the South; in fact, I know it is not followed very generally. If the temperature rises, which will most commonly be the case in a few hours, put it back in the bath, and go through the process again; and I know of nothing that will keep the little patient with hot fever so comfortable and that will so aid us in bringing it safely through to health as this simple means.

